# Supplementary material for: Evaluation of a biomarker for amyotrophic lateral sclerosis derived from a hypomethylated DNA signature of human motor neurons
Source: BMC Med Genomics. 2025 Jan 14;18:10. doi: 10.1186/s12920-025-02084-w (PMC11734586; doi:10.1186/s12920-025-02084-w)
Supplement: Supplementary file 2 — Supplementary Material 2 [file 12920_2025_2084_MOESM2_ESM.pdf]

Supplementary Figures and Tables:

Supplementary Figure 1: Methodological pipeline for identification and testing of a motor neuron (MN) specific methylation signature as a biomarker for ALS. We performed WGBS of DNA derived from iPSC-derived human motor neurons (MN) which we used to derive methylation blocks encompassing the entire genome. To verify that this profile was representative of human CNS neurons we compared the methylation pattern across all blocks with similar blocks derived from an atlas of all cell-types and tissues [5]. We demonstrated that the pattern of methylation derived from iPSC-derived MN clustered together with other CNS neurons, and that genes associated with these methylation blocks were consistent with the transcriptome of human MN. Next, we isolated a set of hypomethylated regions which was specific to MN, to allow detection of DNA released from dying MN. To aid the choice of regions we created a set of synthetic mixes of WGBS reads from different cell types, including MN DNA at different concentrations. These synthetic mixes enabled us to simulate cfDNA, which is derived from a mix of tissues, and to determine which regions were optimal for detection of MN DNA at the lowest possible concentration. Finally we tested our MN specific biomarker in real WGBS from plasma cfDNA. We deconvoluted the cfDNA methylation profile to determine the relative proportions of contributing cell types, including MN.

Supplementary Figure 2: Deconvolution of plasma and CSF cfDNA WGBS data to detect MN-specific methylation signatures. (A) AUC for detection of motor neuron derived cfDNA, refers to **Figure 4B-C. (B)** To estimate the likelihood of false-positive detection we determined the percentage of healthy cfDNA plasma samples where each expected cell-type is identified across all sets of marker regions and coverage. Only two cell types are observed in every sample, whilst all cell types are identified in at least one sample. **(C)** Measurement of the proportion of CNS neuron-specific and MN-specific MN within CSF cfDNA. CNS neuronal and oligodendrocyte DNA is detectable within CSF cfDNA derived from four patients with hydrocephalus.

Supplementary Table 1: iPSC-derived motor neuron whole genome bisulfite sequencing. Details of lines are provided together with QC from WGBS per technical replicate.

Supplementary Table 2: Genomic regions and CpG sites specifically hypomethylated in iPSC-derived motor neurons compared to other cell types. P-value indicates the comparison of methylation with other cell-types within a methylation atlas [5] via a one-sided

t-test. Genomic coordinates are Genome Reference Consortium Human Build 38 (GRCh38).

Supplementary Table 3: Sequencing coverage in WGBS of CSF cfDNA samples.

Supplementary Table 4: Software used in "Evaluation of a biomarker for amyotrophic lateral sclerosis derived from a hypomethylated DNA signature of human motor neurons"
