# Supplementary figures and images for: Evaluation of a biomarker for amyotrophic lateral sclerosis derived from a hypomethylated DNA signature of human motor neurons
Source: BMC Med Genomics. 2025 Jan 14;18:10. doi: 10.1186/s12920-025-02084-w (PMC11734586; doi:10.1186/s12920-025-02084-w)

**A**

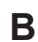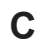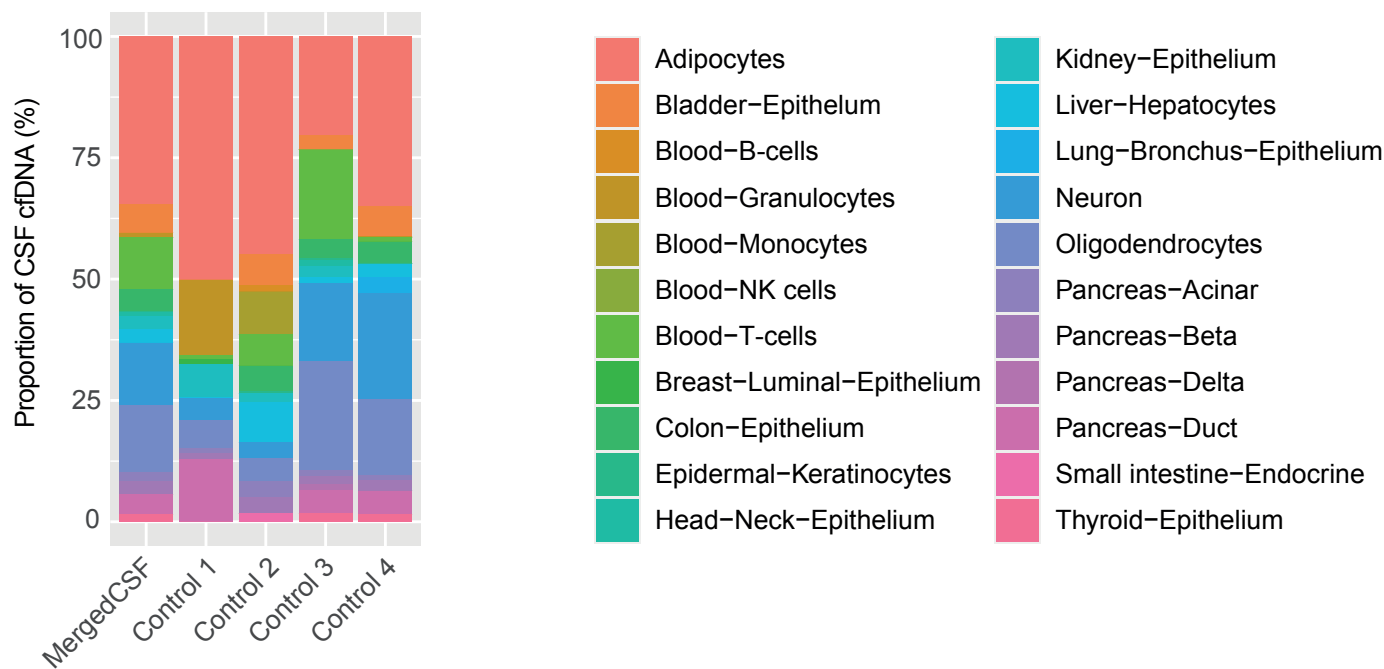

Supplement: Supplementary file 3 — Supplementary Material 3 [file 12920_2025_2084_MOESM3_ESM.pdf]

Supplementary Figure 1

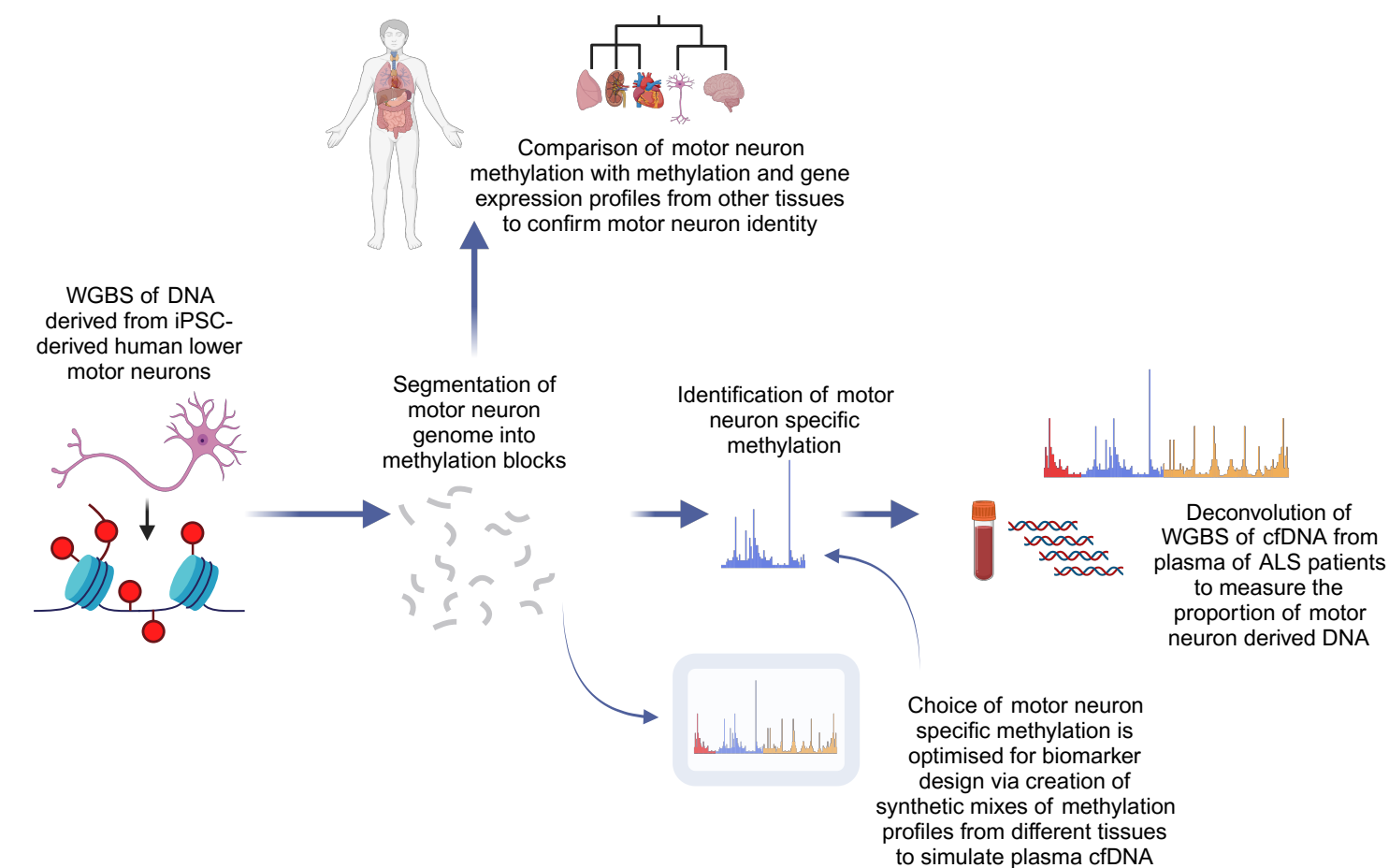

Supplement: Supplementary file 4 — Supplementary Material 4 [file 12920_2025_2084_MOESM4_ESM.pdf]
